# Effects of Near-Infrared Pulsed Light on the Attention of Human Beings Using Electroencephalography

**DOI:** 10.3390/life13071490

**Published:** 2023-06-30

**Authors:** Jih-Huah Wu, Chia-Yen Yang, Yang-Chyuan Chang, Yi-Chia Shan

**Affiliations:** 1 Department of Biomedical Engineering, Ming Chuan University, No. 5, Deming Rd., Gweishan Township, Taoyuan 333, Taiwan; cyyang@mail.mcu.edu.tw; 2 Department of Neurology, Min-Sheng General Hospital, No. 168, Jin-Kuo Rd., Taoyuan 330, Taiwan; ycchang@e-ms.com.tw; 3 Department of Information and Telecommunications Engineering, Ming Chuan University, No. 5, Deming Rd., Gweishan Township, Taoyuan 333, Taiwan; ycshan@mail.mcu.edu.tw

**Keywords:** attention, EEG, brainwave, photobiomodulation

## Abstract

In our previous studies, photobiomodulation (PBM) stimulation can induce significant brain activation in normal subjects. In an open-eye study, the PBM stimulation was able to increase the power of alpha rhythms and theta waves, as well as decrease the beta activities after PBM stimulation. However, in the closed eyes study, the alpha rhythms in the laser group were reduced. This means the PBM stimulation can induce specific brainwaves under different conditions. Thus, to investigate the effects of PBM stimulation on human’s attention, forty students were recruited in this single-blind randomized trial. A PBM stimulator, with seven pcs laser diodes (LDs), frequency 10 Hz, 30 mW/each LD, and wavelength 830 nm, was used to radiate the palm of the subject. PBM stimulation was found to induce significant variation in beta activity in most of the regions of the brain in the laser group. Compared to the placebo group, the PBM stimulation has a significant change in beta activity on electroencephalography (EEG). Three types of tests, the random number test, the Stroop color-word test, and the Multiple-Dimension Attention Test (MDAT), were used to evaluate the effects of the PBM stimulation. The scores of MDAT in the laser group increased more significantly than those in the placebo group after PBM stimulation (*p* < 0.01). An improvement in attention was observed in this study.

## 1. Introduction

Attention is a limited cognitive resource that selectively concentrates on a discrete aspect of information while ignoring others. It is typically divided into two types: active and passive, also known as top-down and bottom-up, or endogenous and exogenous [1]. Active attention is the ability to consistently maintain mental effort on what is important at a given moment and to inhibit irrelevant objects. For example, when you are watching a favorite show and someone asked you to wash the dishes, you may not hear it. Passive attention is the involuntary process directed by external events that stand out from the environment, such as people talking, cars driving by, bright flashes of light from a cell phone, and a multitude of other events that are picked up by the senses. Attention is an important mental process that affects memory and learning performance. Hence, children, adults, and even disorder patients benefit from trainings that improve their attention [2].

Several studies have found that trainings improved the attention and cognitive performance of subjects, ranging from children to young adults to patients with attention deficit hyperactivity disorder (ADHD) [3,4]. For example, Jiang et al. [5] summarized current success in the real-time neurofeedback training of older brains, aiming to match those of younger brains in tasks that require attention or working memory. Those trainings utilized traditional electroencephalography (EEG) and new EEG neuro-markers, including relative power of theta, alpha, beta, theta/alpha and theta/beta ratios, in children with ADHD and learning disorders. Furthermore, Knowles and Wells [6] used tests to investigate whether attention control components of the attention training techniques elicit a specific signal that is different from passive listening. They recruited 36 healthy volunteers that were randomely assigned to the active or control groups. They then calculated their EEG power of the theta, alpha, and beta bands during the resting state before and after training. Their results suggest that a single dose of attention training increases alpha and beta oscillations in the frontoparietal networks associated with top-down attentional or executive control.

The brainwave rhythm is associated with physical and mental states. The most dominant patterns of change in patients with ADHD, schizophrenia, and obsessive-compulsive disorder (OCD) are power increases across lower frequencies (delta, 1–4 Hz and theta, 4–8 Hz) and decreases across higher frequencies (alpha, 8–13 Hz; beta, 13–30 Hz and gamma, 30–50 Hz) [7]. If we can induce specific brainwaves by using stimulators, the subjects’ attention and even the disorders mentioned above may be improved. Low-frequency stimulation (1 to tens of Hz), including electricity, sound, magnetic field, and light, is widely used in different areas of medicine [8]. The EEG activity can be affected by different stimulation modalities [9,10,11,12], including visual, auditory, and somatosensory stimulation. From our previous studies [13,14], the insensible low-level light (LLL) (also known as photobiomodulation (PBM)) stimulation on the palm can evoke EEG. In the open-eye study, we found a significant increase in the subjects’ alpha rhythm and theta waves, mainly in the posterior head regions. The amplitude power of beta activities in the anterior head regions decreased after PBM stimulation [13]. However, in a closed-eye study, we found a drop of alpha rhythm in the posterior part of the brain in the beginning and alpha diffusion in the frontal part of the brain [14]. Thus, whether the subjects’ eyes are open, specific brainwaves can be induced with the same stimulator operated in 10 Hz frequency. The goal of this study is to investigate whether attention can be improved with a similar stimulator.

## 2. Materials and Methods

### 2.1. Participants

Prior to the trial, the study protocol was approved by the Institutional Ethics Committee of Min-Sheng General Hospital. Each participant was required to give written informed consent. This study was directed according to the guidelines in the Helsinki Declaration. Forty healthy university students were recruited. The subjects were randomly assigned to two groups: 20 testers in the laser group (irradiated by the stimulator: wavelength of the laser, 830 nm; seven laser diodes (LDs); output power 30 mW/each LD; frequency 10 Hz) and 20 in the control group (sham laser: the lasers had not been activated). Information regarding the subjects in this study is shown in Table 1. Statistical analysis showed no significant difference between the two groups for age (*p* = 0.260). Exclusion criteria included (a) having a history of psychiatric disorders, for example, major depression, substance abuse, schizophrenia, or paranoid disorder; (b) having cardiopulmonary disease; and (c) receiving medication.

### 2.2. PBM Stimulator

**Figure 1:**
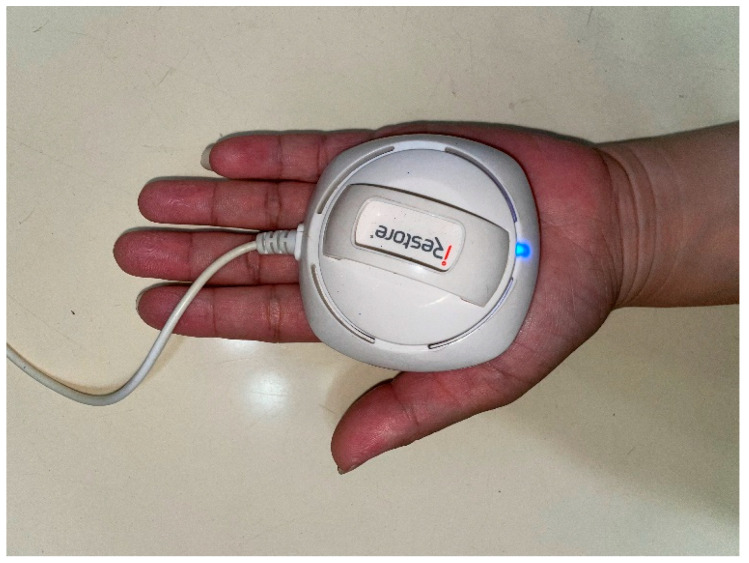
The PBM stimulator was applied to the palm of the tester.

In this study, a PBM stimulator (iRestore Multi-Channel Laser Therapy System, Jin-Ciang Technology, Taoyuan, Taiwan) was applied to the palm of the tester, as shown in Figure 1. The output head has seven light spots. The application mode of the laser instrument was an output frequency of 10 Hz and a wavelength of 830 nm. Each output power of a laser diode is 30 mW, and the total dose is 63 J. Due to low output power (30 mW) of LD, the PBM stimulation is insensible and does not have a thermal effect.

### 2.3. Procedure

**Figure 2:**
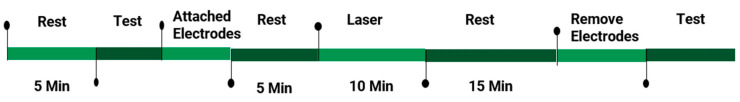
The protocol of this study.

The study used a single-blind randomized trial. The subjects were unaware of the groups they were in. Each subject sat in an arm-chair and was then required to put his or her left palm on the PBM stimulator or hold the stimulator. Participants were instructed to relax, follow the open-eyes directive, and refrain from making any movements. In the laser group, the laser diodes were turned on for 10 min and turned off in the control group. In the beginning, the testers were asked to relax for five minutes in order to stabilize physiological parameters. The ongoing EEG was then recorded with eyes opened in three stages (six sessions): before stimulation (baseline 5 min, session 1), during stimulation (laser stimulation, 10 min, session 2 and session 3), and after stimulation (post-stimulation, 15 min, and session 4, 5, and 6), as shown in Figure 2.

### 2.4. Control

The low-power near-infrared laser is ideally suited for a single-blind study, since the laser light is invisible and emits no heat or any other detectable indication. The testers were randomly divided into two operational modes: a laser group, which received the real laser stimulation, and a control group, which received no laser stimulation. The control group had the same procedure as the laser group, but the laser stimulator was not turned on.

### 2.5. EEG Recording and Measurement

Raw EEG data were collected with the aid of an amplifier (NeuroScan-NuAmps, NeuroScan, Charlotte, NC, USA) from 32 recording sites according to the international 10–20 system (Fp1, Fp2, F3, F4, F7, F8, Fz, FT7, FT8, FC3, FC4, FCz, A1, A2, T3, T4, C3, C4, Cz, TP7, TP8, CP3, CP4, CPz T5, T6, P3, P4, Pz, O1, O2, and Oz) using sintered Ag–AgCl electrodes. The left and right mastoids (A1, A2) were used as an averaged ear reference for recording offline, with a FPz as the grounding electrode. The signals were filtered by a bandpass filter from 0 to 260 Hz and digitized at a sampling rate of 1000 Hz. The average reference was used for all channel recordings with impedances below 10 kΩ.

All processing steps were executed by an in-house program written in MATLAB (MathWorks, Natick, MA, USA). The EEG signals were preprocessed according to the following three steps. First, whole signals were detrended to remove means, offsets, and slow linear drifts over the course of time. Second, the detrended signals were filtered using a 0.5–50 Hz bandpass filter (pop_eegfiltnew.m from EEGLAB toolbox). Finally, the filtered signals were decomposed using the FastICA algorithm to manually remove components containing artifacts in the form of eye movements, blink artifacts, and electrocardiogram activity. After preprocessing, the data were then segmented into six epochs (i.e., rest0, stimulus1, stimulus2, rest1, rest2, rest3), with five minutes each epoch. We then calculated the relative power in each band (theta, 4–8 Hz; alpha, 8–13 Hz; beta, 13–30 Hz; and gamma, 30–50 Hz), divided by the sum of the power across the entire frequency range from 0.5 to 50 Hz.

A two-sample Student’s *t*-test was used to compare the EEG power in each band between the laser group and the control group, with statistical significance defined as *p* < 0.05.

### 2.6. Attention Test

The clinical model of attention was proposed by Sohlberg [15]. It was divided into five dimensions: Focused Attention, Selective Attention, Sustained Attention, Alternating Attention, and Divided Attention. To increase the diversity of the attention tests, we designed three types of tests. In this study, a random number test, the Stroop color–word test [16], and the multiple-dimension attention test (MDAT), were used for evaluating the attention performance in the laser group and the placebo group. In the random number test, Excel was used to produce random number sequences 0 to 9, and the amount of each number ranges from 50 to 80. The subject searches for and marks the specified number during the test. Additionally, the subject was required to search only once. Each subject must concentrate on finding the correct number in this test, which takes about 1 to 3 min. The purpose of the random number test is to assess the performance of focused attention.

In the Stroop color–word test, we use a mismatch between the name of a color and the color (e.g., the word “blue” printed in white ink) extensively to evaluate the subject’s ability to inhibit cognitive interference. A total of 50 questions were designed, which involved the interference of similar colors, such as background and pattern. Each subject must quickly identify the color of the word, which is assigned randomly. It takes about 0.8 to 1.3 min for each subject to finish the test. However, it will take longer for the subject to name the color of the word. The purpose of the Stroop color–word test is to assess the performance of selective attention.

In the MDAT, the questions include single choice questions, multiple choice questions, and blank filling questions. Each subject must remember the details of 15 pictures, and the subject has 7 s for each picture. After that, the subject will answer the 10 questions (10 points per question) in this 3-min test. The purpose of the MDAT is to assess the performance of focused attention, selective attention, sustained attention, alternating attention, and divided attention.

## 3. Results

### 3.1. EEG Results

**Figure 3:**
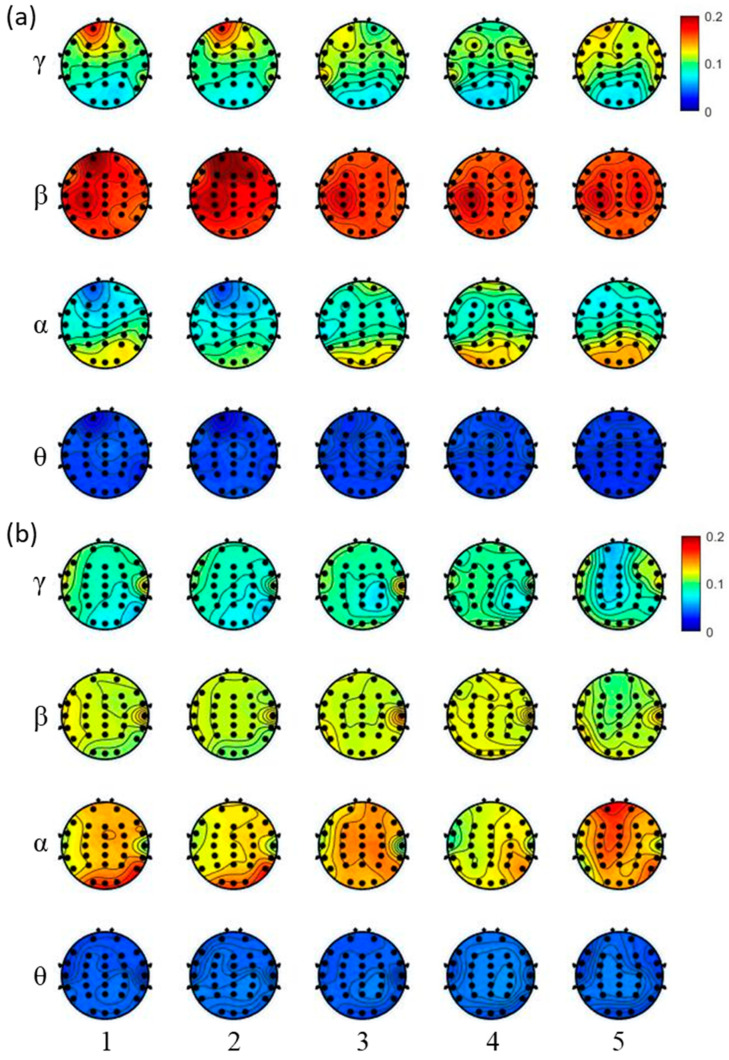
Representative topographic maps of the power ratios in the laser (**a**) and the control (**b**) groups. The number represents the stage order of the epochs after rest0, i.e., 1 indicates stimulus1, 2 indicates stimulus2, 3 indicates rest1, 4 indicates rest2, and 5 indicates rest3.

Figure 3 shows the topography maps of the relative power in different frequency bands from one of the subjects in the groups. The number represents the stage order of the epochs after rest0, i.e., 1 indicates stimulus1, 2 indicates stimulus2, 3 indicates rest1, 4 indicates rest2, and 5 indicates rest3. Compared to the control group, the beta band power was higher, and the alpha band power was lower in the laser group. In the laser group, there was also a greater focus around the frontal area in the beta and the gamma bands, especially during stage 1 and stage 2. Additionally, there was a greater focus around the occipital area in the alpha band. In the control group, the distributions were more dispersed around the frontal, central, parietal, and occipital areas in the alpha band.

**Figure 4:**
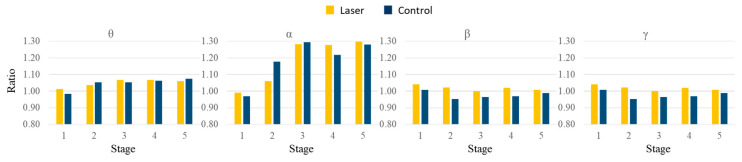
Mean values of the ratios from the whole brain in the theta, alpha, beta, and gamma bands across all subjects. The numbers correspond to the time stages.

**Figure 5:**
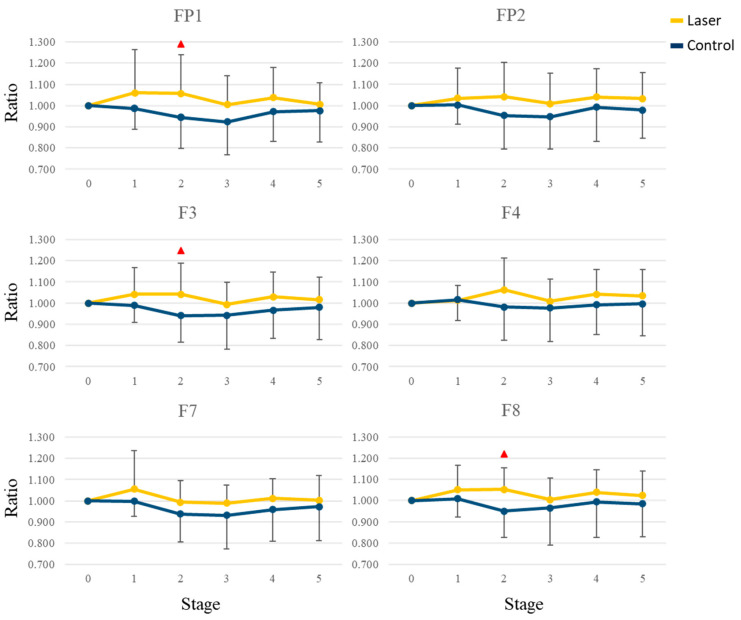
The mean and standard deviation values of the ratio in the beta band for six frontal electrodes. The red triangle indicates a significant difference (*p* < 0.05) between the laser and the control groups.

**Figure 6:**
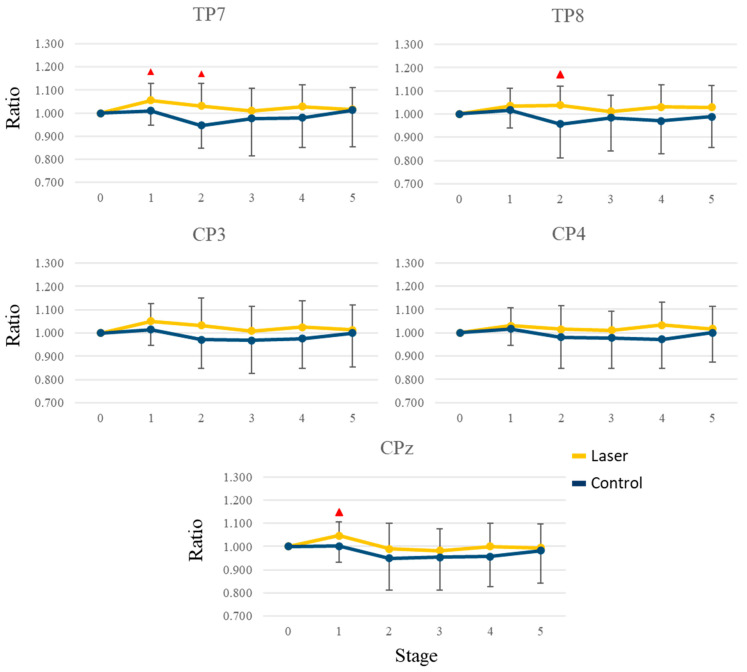
The mean and standard deviation values of the ratio in the beta band for five parietal electrodes. The red triangle indicates a significant difference (*p* < 0.05) between the laser and the control groups.

We then used the first epoch, i.e., rest0, as the base condition and divided the relative power in each epoch with that in the first epoch. We calculated the average ratios of the whole brain in different frequency bands across all subjects. Figure 4 shows the mean values in the five stages. When comparing the two groups, there was a decrease in the alpha band during stage 2, whereas there was an increase in the beta band after stage 2. Besides, the ratios were higher in the laser group, especially in the beta and gamma bands. To uncover the difference, we compared the ratio for each electrode at every stage. In the theta and alpha bands, no significant difference was found. In the beta band, there were significant differences at FT7, TP7, and CPz (*p* = 0.043, 0.046 and 0.038) during stage 1 and at FP1, F3, F8, FT7, FC3, FT8, T3, T4, TP7, TP8, and T5 (*p* = 0.042, 0.029, 0.010, 0.016, 0.045, 0.001, 0.003, 0.003, 0.011, 0.042, and 0.049) during stage 2. In the gamma band, there was a significant difference (*p* = 0.030) at FP1 during stage 2. Figure 5 and Figure 6 show the mean and standard deviation values of the ratio in the beta band at 6 frontal and 5 parietal electrodes, respectively. After stage 3, the differences became small and insignificant.

### 3.2. Attention Test Results

A two-tailed paired *t*-test was applied to compare the difference between two groups.

**Figure 7:**
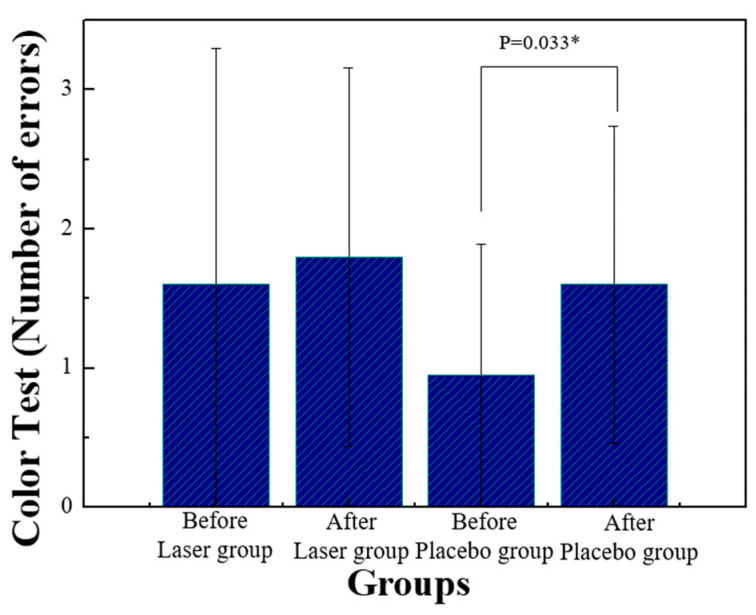
The statistical analysis of the Stroop test by comparing the error values before and after the stimulation in either group. * *p* < 0.05 by paired-sample *t*-test.

All the statistical analyses were executed with the SPSS software (version 11). A statistical significance was recognized as *p*-value < 0.05. The errors of the Stroop test before and after, in the placebo group, increased significantly, as shown in Figure 7.

**Figure 8:**
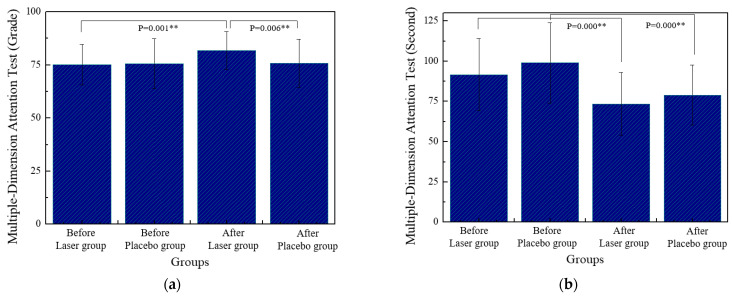
The statistical analysis of the MDAT by comparing the values of grade (**a**) and time (**b**) in two groups. ** *p* < 0.01 by paired-sample *t*-test.

The grades of MDAT before and after stimulation in the laser group increased significantly. There are also significant differences between the laser group and the placebo group after stimulation, as shown in Figure 8a. In either group, the time of MDAT before and after the stimulation is significantly reduced, as shown in Figure 8b.

## 4. Discussion

### 4.1. Light Stimulation and EEG

Tsai and Hamblin observed that there were phasic inhibitory and excitatory afferent responses when the sensory epithelium was irradiated by various types of infrared light with pulses [17]. From our studies [13,14], specific brainwaves can be induced by the somatic stimulation with near infrared light. In 2011, we used laser acupuncture stimulation on the left foot’s Yongquan (KI1) acupoint and verified the effect by functional magnetic resonance imaging analysis. The results showed that the primary motor cortex and middle temporal gyrus of the left hemisphere and bilateral cuneus in the 10 Hz-modulated group found significant activations [18]. Moreover, the photic stimulation with frequency could induce regulatory functions in the brain. H. Sakamoto and co-workers showed that intermittent photic stimulation at 10 Hz could externally induce a coherent state in the cortex and persist as a form of short memory [19]. Milone’s group showed that a 10 Hz red light-emitting diode (650 nm) light, consistent with the alpha rhythms, gives rise to cognitive function and regulatory function in the brain [20]. Specific brainwaves and functions of the brain can be induced with different kinds of light stimulations.

### 4.2. EEG and Attention

The two groups in our study had been tested with three kinds of attention tests before the PBM stimulation. In the present study, the main EEG changes induced by this insensible PBM stimulation at the palm included the increase of beta rhythms in the front and parietal lobes in the laser group, see Figure 4 and Figure 5, sessions 1 and 2. It means that the beta brainwave (induced by a series of tests) can be sustained with 10 Hz stimulation. From our previous studies, the alpha rhythms in the laser group were induced in the open-eyes study [13]. However, with the same stimulation, the alpha rhythms in the laser group were reduced in the closed eyes study [14]. This mean that the PBM stimulation can induce different brainwaves under different conditions—closed-eye or open-eye. According to the attention tests, the brainwaves in the laser group are dominant at the beta band at many sites, even if the subjects were stimulated with 10 Hz frequency (in the alpha band). We summarize the EEG results of our previous studies [13,14,21] and this study in Table 2.

In a test condition in which a subject has to perform some kind of tasks, alpha desynchronizes [22], but theta synchronizes [23,24]. The findings [25,26] document that theta varies as a function of alpha frequency and suggest using alpha frequency as a common reference point for adjusting different frequency bands. EEG alpha and theta oscillations reflect cognitive and memory performance [27]. A single dose of attention training increases alpha and beta oscillations in the frontoparietal lobe [6]. More attention tests in this study, therefore, increase beta waves.

### 4.3. Methods to Improve Attention

Recently, most research has been focused on some training methods to improve students’ attention, such as computer games [3], meditation training [4], neurofeedback [5,28], virtual and augmented reality [29,30], and music [31]. Most of these methods take a long time to improve the attention of the testers. For example, EEG neurofeedback training was applied to children with ADHD, and cognitive functions (sustained attention, verbal working memory, and response inhibition) were improved in the treatment group [28]. Lai and Chang used fixation focus training activity to improve the attention of elementary school students [32]. The experiment group underwent focus training, conducted once a week, for 12 weeks. There were significant differences in the total scale, focused attention, and selective attention. However, it takes 12 weeks [32] or more [28] to improve the attention of the subjects. From the experimental results, specific brainwaves of the subjects can be induced by ten-minute stimulation. The purpose of the Stroop color–word test is to assess the performance of selective attention. The errors in the Stroop test before and after in the placebo group significantly increased, as shown in Figure 7. The beta bands of brainwaves in the placebo group have not been induced or enhanced. The performance of MDAT has been improved in the laser group, as shown in Figure 8a. On the other hand, the time of MDAT in the two groups is significantly reduced before and after the test, as shown in Figure 8b. Such a reduction was caused by his or her familiarity with the test when the subject retakes the test. The purpose of the MDAT is to assess the performance of focused attention, selective attention, sustained attention, alternating attention, and divided attention. It means the subjects need more attention when they conduct the MDAT test.

### 4.4. Limitations and Future Directions

The major limitation of the study is that the present study was conducted in a healthy population. The gender and age are also the parameters that affect EEG in different situations. The changes in EEG activity were used to detect a driver’s drowsiness [33] and fatigue [34] for different genders. The age and gender parameters will be considered in future work. Additionally, staying up late significantly affects brainwaves, and thus future studies might try to capture more detailed and more objective indicators of sleep quality. On the other hand, brainwave rhythm is associated with mental states. For example, the most dominant patterns of EEG change in patients with ADHD, schizophrenia, and OCD are power increases across lower frequencies (delta and theta) and decreases across higher frequencies (alpha, beta, and gamma) [7]. If we recruited the patients who have these mental problems in this study, the experimental results would be different.

Moreover, the effects caused by light stimulation on the palm are not limited to inducing specific brainwaves. For example, the blood circulation of the brain and the lungs (according to traditional Chinese medicine, the pericardium and lung meridians are located on the thumb and middle finger) may be enhanced. This affects the rate of oxygen consumption in the brain. Rojas et al. verified that LLL therapy can increase the rate of oxygen consumption in the prefrontal cortex in vivo. Additionally, they showed that LLLT-treated rats had an enhanced extinction memory as compared to controls [35]. The effects of PBM on the brain via somatic stimulation deserve further study.

## 5. Conclusions

The effects of PBM stimulation at the palm on attention were investigated. This study applied PBM stimulation to university students to induce changes in EEG power and improve attention. The specific brainwaves can be induced with the PBM stimulator operated at a 10 Hz frequency. The power of beta and gamma bands increased in many sites in the brain regions. The scores of MDAT in the laser group increased significantly. We believe the results in this study have practical implications for the medical field. For instance, the long-term use of light stimulation may have potential applications in several disorder types, including ADHD, schizophrenia, depression, and OCD.

## Figures and Tables

**Table 1:** Subject Information.

Item	Experimental Group	Placebo Group
Subject number	20	20
Male/female	12/8	12/8
Age (years)	21.35 ± 0.74	21.78 ± 1.16, *p* = 0.260

**Table 2:** Significant variations in the normalized power in the brain region with a similar light stimulator.

Stimulator		Laser (30 mW)	LED (30 mW) [21]	Laser (7 mW) [13]	Laser (7 mW) [14]
Task		Conducting 3 Tests (Open Eyes)	No Action (Open Eyes)	No Action (Open Eyes)	No Action (Closed Eyes)
Brainwave	Beta	FT7, TP7 and CPz; FP1, F3, F8, FT7, FC3, FT8, T3, T4, TP7, TP8 and T5 increase ↑	C4-P4 increase ↑	T3-T5,T4-T6, F7-T3, F8-T4 decrease ↓	P3, O1, P4, O2 decrease ↓
	Alpha	None variation	C3-P3, C4-P4, P3-O1, P4-O2, T3-T5, T4-T6 more increase ↑↑	C3-P3, C4-P4, P3-O1, P4-O2, T3-T5, T4-T6 increase more increase ↑↑	P3, O1, P4, O2 F3, F4, C3, C4 more decrease ↓↓
	Theta	None variation	P4-O2, T4-T6, T3-T5 increase ↑	P3-O1, P4-O2, T4-T6increase ↑	None variation
	Delta	None variation	None variation	None variation	None variation

## Data Availability

The data used to support the findings of this study are included within the article.
